# Cutaneous cryptococcosis resembling keratoacanthoma

**DOI:** 10.1590/0037-8682-0300-2024

**Published:** 2025-07-07

**Authors:** Alex Panizza Jalkh, Naira Sulany Oliveira de Sousa, Juan Diego Ribeiro de Almeida, Flávia da Silva Fernandes, Kátia Santana Cruz, Hagen Frickmann, Marcus Vinícius Guimarães Lacerda, João Vicente Braga de Souza

**Affiliations:** 1 Fundação de Medicina Tropical Dr. Heitor Vieira Dourado, Manaus, AM, Brasil.; 2 Programa de Pós-Graduação em Biodiversidade e Biotecnologia da Rede BIONORTE, Manaus, AM, Brasil.; 3 Instituto Nacional de Pesquisas da Amazônia, Manaus, AM, Brasil.; 4 Department of Microbiology and Hospital Hygiene, Bundeswehr Hospital of Hamburg, Hamburg, Germany.; 5 Institute for Medical Microbiology, Virology and Hygiene, University Medicine Rostock, Rostock, Germany.; 6 Instituto de Pesquisas Leônidas & Maria Deane, Fiocruz, Manaus, AM, Brasil.; 7 University of Texas Medical Branch, Galveston, USA.

**Keywords:** Cryptococcus, Cutaneous cryptococcosis, keratoacanthoma

## Abstract

Cutaneous cryptococcosis is a manifestation of systemic cryptococcal infection, characterized by polymorphic skin lesions that can make diagnosis challenging. We report an HIV-positive patient with a solitary facial nodule, initially diagnosed as keratoacanthoma. Histopathology, fungal culture, and cerebrospinal fluid analysis confirmed disseminated cryptococcosis with central nervous system involvement caused by *Cryptococcus neoformans* genotype VNI. He was treated with amphotericin B followed by fluconazole, resulting in complete clinical resolution. This case underscores the need to consider disseminated cryptococcosis in the differential diagnosis of tumorlike skin lesions, particularly in HIV-positive patients.

## INTRODUCTION

Cutaneous cryptococcosis is a manifestation of systemic fungal infection, particularly affecting immunocompromised individuals, such as those with human immunodeficiency virus/acquired immunodeficiency syndrome (HIV/AIDS)^1^. Its polymorphic skin presentations, including papules, nodules, plaques, and ulcerations, often mimic other dermatological conditions, leading to potential diagnostic delays^1^ ^,^ ^2^. Among these, tumor-like lesions may pose a specific challenge because of their resemblance to neoplastic processes^2^ ^-^ ^4^.

Keratoacanthoma is a low-grade skin tumor characterized by a solitary dome-shaped lesion with central keratin-filled craters, commonly found in sun-exposed areas^5^. Comprehensive research is available in major scientific databases, including the Cochrane Library, LILACS, SciELO, MEDLINE, PubMed, and PMC (PubMed Central). No published reports have described cryptococcosis clinically resembling keratoacanthoma.

This case report presents an HIV-positive patient with a facial lesion initially presumed to be keratoacanthoma. However, histopathological and microbiological findings revealed disseminated cryptococcosis caused by *Cryptococcus neoformans* genotype VNI, with cutaneous and central nervous system involvement. This case demonstrates the importance of considering disseminated fungal infections in the differential diagnosis of atypical skin nodules in immunosuppressed patients.

## CASE REPORT

**FIGURE 1:**
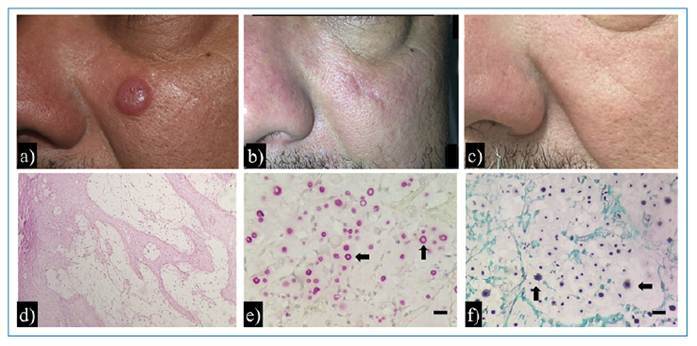
Clinical presentation, histopathological findings, and long-term outcome in a case of cutaneous cryptococcosis mimicking keratoacanthoma. Clinical presentation: **(a)** Firm, rounded, skin-colored nodule on the patient’s face, with well-demarcated borders and a central keratinous crust. **(b)** Postoperative aspects six months after lesion excision and antifungal treatment. **(c)** No signs of recurrence observed after 10 years of clinical follow-up. Histopathological findings: **(d)** Hematoxylin and eosin (H&E) stain showing a dense dermal inflammatory infiltrate composed predominantly of lymphocytes, histiocytes, and epithelioid cells (original magnification ×100). **(e)** Mucicarmine stain highlighting thickened polysaccharide capsules of yeast-like fungal cells (original magnification ×400; scale bar = 10 µm). **(f)** Grocott-Gomori’s methenamine silver (GMS) stain revealing numerous oval fungal structures with thick, dark-stained cell walls within the dermis (original magnification ×400; scale bar = 10 µm).

A 46-year-old man living with HIV visited the dermatology outpatient clinic of a reference institution with a facial lesion that he considered aesthetically displeasing (Figure 1A). The lesion had developed approximately two weeks prior to consultation. He denied any history of local skin trauma, fever, headache, or other significant symptoms. The patient lived in Manaus (Brazilian Amazon) and worked as an air conditioning cleaner. His comprehensive medical history included treatment for pulmonary tuberculosis and a long-standing 11-year antiretroviral regimen (tenofovir disoproxil fumarate, lamivudine, and ritonavir-boosted darunavir). He was also receiving treatment for obesity, dyslipidemia, grade I hepatic steatosis, systemic arterial hypertension, and biliary lithiasis.

Despite ongoing antiretroviral therapy, laboratory tests performed at consultation showed a critically low CD4+ lymphocyte count of 56 cells/mm³ and a viral load of 5,000 copies/mL. This severe immunosuppression was potentially due to non-compliance with HIV treatment, likely exacerbated by the self-reported use of alcohol, marijuana, and cocaine. Additionally, he was not a regular patient and frequently missed appointments, which undoubtedly contributed to challenges in managing his condition. 

During physical examination, a firm, elastic nodule was observed on the patient’s face, measuring approximately 1.5 cm×1.5 cm×1.0 cm. The lesion was located in a sun-exposed area, a common site for actinic damage and keratinocytic neoplasms. It appeared as a rounded, firm, skin-colored nodule with well-defined borders and a central crust. These features are hallmark characteristics of keratoacanthoma, and the initial clinical impression favored this diagnosis^5^.

Based on this diagnostic hypothesis, an excisional biopsy of the lesion was performed (Figure 1B and 1C ), and the specimen was sent for histopathological examination. The procedure was performed on the same day as the dermatological consultation, and a histopathological diagnosis was made 30 days later. Initial histopathological analysis was performed using hematoxylin and eosin (H&E) staining, which revealed inflammatory infiltration of the dermis (Figure 1D) composed of lymphocytes, histiocytes, and epithelioid cells. Subsequently, Mucicarmine of Meyer (MM) and Grocott-Gomori methenamine silver (GMS) stains were used, revealing numerous oval fungal structures with thickened capsules, suggestive of *Cryptococcus* spp. (Figure 1E and 1F). With the histopathological findings, pulmonary (chest tomography) and neurological (cranial computed tomography [CT] and cerebrospinal fluid examination) evaluations were performed. Chest CT revealed subpleural emphysematous bullae in the right upper lobe, pleural thickening, air space consolidation with air bronchograms, and a small cavitary lesion. 

Direct examination of cerebrospinal fluid using Nankin ink revealed the presence of encapsulated yeast, a hallmark of *Cryptococcus* infection. The specimen was cultured on Sabouraud dextrose agar, which confirmed fungal growth consistent with *Cryptococcus* species-specific colony morphology. Two phenotypic tests were performed to identify the species complex: the phenol oxidase test, which was positive, indicating melanin production by *Cryptococcus neoformans*, and the canavanine-glycine-bromothymol blue (CGB) agar test, where no growth or color change was observed, further supporting the identification of *C. neoformans*. DNA was extracted from fungal colonies using the phenol-chloroform method, followed by PCR amplification of a URA5 sequence fragment using primers described by Meyer et al.^6^ ^,^ ^7^. The amplicons were digested with the restriction enzymes *HhaI* and *Cfr13I* (Sau96I), and the restriction pattern was visualized using 3% agarose gel electrophoresis. Banding patterns were compared with reference strains representing molecular types VNI-VNIV and VGI-VGIV, confirming that the isolate was *Cryptococcus neoformans* molecular type VNI^8^. These microbiological findings, combined with histopathological evidence and neurological manifestations, confirmed the diagnosis of disseminated cryptococcosis.

Given this situation, the patient was hospitalized and treatment with amphotericin B deoxycholate was initiated and administered for one month, reaching a cumulative dose of 2 g. Following this initial phase, fluconazole 400 mg/day was initiated and maintained for 24 weeks. Subsequently, fluconazole 150 mg/week was initiated, a regimen the patient continues to follow till date due to HIV positivity. With the management of cryptococcal meningitis, the patient continued antiretroviral therapy (ART), which was effective in maintaining controlled HIV viral loads throughout the follow-up period. No signs of recurrence were detected over a 10-year follow-up.

## DISCUSSION

This report describes a rare and diagnostically challenging presentation of cutaneous cryptococcosis mimicking keratoacanthoma in an immunocompromised, HIV-positive patient. The facial lesion, which was initially suspected to be a keratinocytic neoplasm owing to its dome-shaped architecture, central keratin plug, and well-demarcated crateriform border, was confirmed to be of cryptococcal origin. Histopathological examination, microbiological culture, and molecular analysis identified *Cryptococcus neoformans* genotype VNI as the causative agent. To our knowledge, few previous reports describe cryptococcal skin lesions with a striking resemblance to keratoacanthoma, highlighting this case as a novel clinical observation. This unusual presentation prompted further investigations into the pathogenesis and dissemination pathways of the infection, particularly in the context of immunosuppression.

In this case, typical of cryptococcosis in immunocompromised individuals, cutaneous involvement most likely occurred via hematogenous dissemination following the inhalation of fungal propagules^1^. Pulmonary imaging findings were consistent with potential systemic involvement (subpleural emphysematous bullae in the right upper lobe, pleural thickening, airspace consolidation with air bronchograms, and a small cavitary lesion). We detected encapsulated yeasts in the cerebrospinal fluid, confirming systemic dissemination. Similar to previous reports^9^ ^-^ ^12^, disseminated cryptococcosis initially presented with skin lesions even in the “absence” of pulmonary or neurological symptoms.

The atypical appearance of cryptococcal lesions can obscure timely diagnosis, particularly in immunocompromised patients. Along with unusual clinical timelines, the morphological appearance of cryptococcal skin lesions may contribute to diagnostic delays, particularly in immunocompromised hosts^1^. Moreover, these lesions may occasionally have multiple etiologies, including concurrent neoplasia and opportunistic infections. Several reports have described cryptococcal skin lesions mimicking conditions such as squamous cell carcinoma, basal cell carcinoma, and Kaposi sarcoma^1^ ^,^ ^4^ ^,^ ^13^. Grayson et al.^14^ and Ramdial et al.^15^ reported coexisting Kaposi sarcoma and cryptococcosis in patients with AIDS, whereas Pietras et al.^16^ documented a mixed lesion involving *Cryptococcus* spp. infection, Kaposi sarcoma, and *Mycobacterium avium intracellulare* infection. These examples highlight the diagnostic complexity of nodular skin lesions in immunocompromised patients. 

Molecular identification in our case demonstrated *C. neoformans* genotype VNI, aligning with the most prevalent molecular types circulating in northern Brazil^7^. VNI and VGII are the predominant genotypes in this region, with VNI sequence type 93 (ST93) comprising 70-80% of infections in the state of Amazonas^8^. VNI is the most widely distributed genotype globally and the major cause of cryptococcal meningitis in people living with HIV^17^. These findings underscore the likelihood of environmental exposure. 

Following the diagnosis of cryptococcal meningitis, the patient was treated with amphotericin B deoxycholate as induction therapy, followed by maintenance therapy with fluconazole. Antiretroviral therapy was continued to ensure viral suppression. The patient remained recurrence-free over 10 years, demonstrating long-term therapeutic success. Although effective, the applied antifungal regimen differed from the current World Health Organization (WHO) guidelines, which recommend short-course induction using liposomal amphotericin B combined with flucytosine and fluconazole. However, in Brazil’s public healthcare system, liposomal formulations and flucytosine were unavailable during the treatment period. Therefore, clinicians had to rely on deoxycholate amphotericin B, despite its higher nephrotoxicity and infusion-related complications^18^. 

This case study has some limitations. The absence of cryptococcal antigen testing, due to limited availability in the local public health services, hindered timely diagnosis and patient monitoring. Molecular identification was restricted to URA5-RFLP without sequencing or multi-locus sequence typing (MLST), reducing the resolution of the genotyping approach. Although the treatment was successful, the lack of flucytosine and associated nephrotoxicity risk due to deoxycholate amphotericin B application made the therapeutic approach contradictory to international recommendations. Furthermore, there were no complete follow-up data available regarding immune reconstitution control (e.g., CD4+ lymphocyte count recovery), post-treatment cerebrospinal fluid analysis, or imaging assessments during the extended follow-up period.

In conclusion, this case reconfirms the importance of including cryptococcosis in the differential diagnosis of solitary tumor-like skin lesions in immunocompromised individuals, particularly in endemic regions. Prompt surgical excision followed by histopathological and microbiological work-up, was essential for accurate diagnosis and favorable long-term outcomes. These findings underscore the need for high clinical awareness and comprehensive diagnostic assessment to avoid misdiagnosis and improve patient care in settings where *Cryptococcus* is endemic and HIV/AIDS remains prevalent.
